# Deep Learning Renal Segmentation for Fully Automated Radiation Dose Estimation in Unsealed Source Therapy

**DOI:** 10.3389/fonc.2018.00215

**Published:** 2018-06-14

**Authors:** Price Jackson, Nicholas Hardcastle, Noel Dawe, Tomas Kron, Michael S. Hofman, Rodney J. Hicks

**Affiliations:** ^1^Sir Peter MacCallum Department of Oncology, The University of Melbourne, Melbourne, VIC, Australia; ^2^Department of Molecular Imaging, Peter MacCallum Cancer Centre, Melbourne, VIC, Australia; ^3^Department of Physical Sciences, Peter MacCallum Cancer Centre, Melbourne, VIC, Australia; ^4^School of Physics, University of Melbourne, Melbourne, VIC, Australia

**Keywords:** automated segmentation, radionuclide therapy, kidney, nuclear medicine dosimetry, deep learning

## Abstract

**Background:**

Convolutional neural networks (CNNs) have been shown to be powerful tools to assist with object detection and—like a human observer—may be trained based on a relatively small cohort of reference subjects. Rapid, accurate organ recognition in medical imaging permits a variety of new quantitative diagnostic techniques. In the case of therapy with targeted radionuclides, it may permit comprehensive radiation dose analysis in a manner that would often be prohibitively time-consuming using conventional methods.

**Methods:**

An automated image segmentation tool was developed based on three-dimensional CNNs to detect right and left kidney contours on non-contrast CT images. Model was trained based on 89 manually contoured cases and tested on a cohort of patients receiving therapy with ^177^Lu-prostate-specific membrane antigen-617 for metastatic prostate cancer. Automatically generated contours were compared with those drawn by an expert and assessed for similarity based on dice score, mean distance-to-agreement, and total segmented volume. Further, the contours were applied to voxel dose maps computed from post-treatment quantitative SPECT imaging to estimate renal radiation dose from therapy.

**Results:**

Neural network segmentation was able to identify right and left kidneys in all patients with a high degree of accuracy. The system was integrated into the hospital image database, returning contours for a selected study in approximately 90 s. Mean dice score was 0.91 and 0.86 for right and left kidneys, respectively. Poor performance was observed in three patients with cystic kidneys of which only few were included in the training data. No significant difference in mean radiation absorbed dose was observed between the manual and automated algorithms.

**Conclusion:**

Automated contouring using CNNs shows promise in providing quantitative assessment of functional SPECT and possibly PET images; in this case demonstrating comparable accuracy for radiation dose interpretation in unsealed source therapy relative to a human observer.

## Introduction

In comparison to other radiation oncology modalities, personalized dosimetry assessment in unsealed source therapies is relatively uncommon. The process involves the measurement of regional uptake and pharmacokinetics followed by some calculation of radiation transport (1). In the first stage, the concentration of radiopharmaceutical is assessed on imaging and—by collecting a time series or applying known uptake and clearance parameters—an estimate of the number of disintegrations in each tissue is obtained. Finally, decays are converted into radiation absorbed dose through published self- and cross-dose factors or Monte Carlo simulation. Time-activity curve fitting by either least squares or analytical methods is a mechanical process. Similarly, integration of pharmacokinetic data and multiplication of organ or voxel dose factors are trivial mathematical operations. Unfortunately, employing these techniques often requires manual input with a degree of time and expertise that precludes their widespread use. In a previous work, we have demonstrated the feasibility of performing image-based dosimetry to create three-dimensional voxel dose maps (2). This is an automated process that may be applied to any radionuclide treatment where sequential follow-up imaging is available.

The use of neural networks for organ recognition has rapidly surpassed the capabilities of existing automated contouring techniques that rely on either rule-based methods (3) or atlas segmentation (4). Within just a few years the road map for performing pixel-by-pixel segmentation from a practical amount of ground truth data has demonstrated applications across most medical imaging modalities (5–7). These convolutional neural networks (CNNs) are demonstrating utility for image segmentation in CT, MRI, and ultrasound (8, 9). They may be designed to operate based on two-, three-, or even four-dimensional (either time series or multiparametric) images (10, 11). They have shown applications in rapid contouring to offer more efficient radiation therapy treatment planning (12) as well as in the field of computer-aided detection of specific pathologies (13). Moreover, these computational techniques—both inference and model training—are feasible on standard personal computers.

Segmentation of kidney on CT imaging presents challenges because the appearance, particularly at the inferior- and superior-most slices, may closely resemble other abdominal structures in terms of shape and physical density. As such, it is logical to employ a CNN that utilizes 3D kernels across the input volume as a whole (14). The predicted shape on one slice is then informed by features present on subsequent image slices. In this work we employ an automated CNN-based software tool to perform quantitative analysis of SPECT images based on the anatomical outline in a fused CT volume. More specifically, we demonstrate the feasibility of fully automated radiation dose estimation in unsealed source therapy as applied to patients with metastatic prostate cancer treated with radioactive prostate-specific membrane antigen (PSMA).

## Materials and Methods

### Training Image Data

Training cohort was based on a population of manually contoured left and right kidneys from varied group of clinical cases. The largest of these was a set of post-treatment ^177^Lu-octreotate therapy of neuroendocrine cancer acquired on a hybrid SPECT system with low-dose CT acquisition and 5 mm slice thickness (Siemens Symbia T6 & Intevo 16, Siemens Healthineers, Erlangen, Germany). A subset of patients scanned on dedicated diagnostic CT (Siemens Force, 0.8–5.0 mm slice thickness) and radiotherapy simulation CT systems (Philips Brilliance Big Bore, 3 mm slice thickness, Philips Medical Systems, Cleveland, OH, USA) were included to better adapt the model for detection across different populations and equipment types. A total of 89 manually contoured patients were included for training. Each patient was augmented seven times with a random degree of added noise, edge enhancement, Gaussian smoothing, change in global HU values, translation, and in-plane rotation to avoid CNN overfitting due to non-anatomical image feature (6). This provided 712 subjects available for model training. A detailed description of the image augmentation techniques used is given in the Appendix S1 in Supplementary Material.

### Testing Image Data

Independent test images were taken from a cohort of 24 patients involved in a Phase II prospective trial of ^177^Lu-PSMA-617 for treatment of metastatic prostate cancer (ANZCTR12615000912583) (15). Each patient received serial post-treatment quantitative SPECT/CT imaging (16) at timepoints of 4, 24, and 96 h. Three-dimensional radiation dose maps were processed using a previously described technique involving non-rigid image registration, voxel-wise pharmacokinetics analysis, and dose kernel convolution (2). Low-dose, fused CT images were designated as input to the CNN segmentation model. Each kidney in the testing cohort was manually contoured and reviewed by a nuclear medicine physician. Structures were compared to those automatically detected based on dice score, mean distance-to-agreement (per voxel the shortest distance from the surface of one structure to another), volume, and estimated radiation absorbed dose from ^177^Lu therapy according to three-dimensional voxel dose map (17). Mean right and left kidney doses were evaluated for null hypothesis of difference between contour techniques by paired *t*-test.

### Convolutional Neural Network

**Figure 1:**
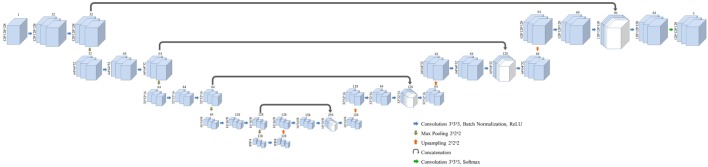
Layer structure and dimensions for the semantic convolutional neural network used in this study. Fully three-dimensional convolutional network structure is adapted from a work by Pazhitnykh et al. (18).

Three-dimensional convolutional neural network was modified from the structure published by Pazhitnykh et al. using 21 convolutional layers (18). CNN architecture was employed with Keras (v2.08) in Python with Tensorflow backend (v1.3) (19). A dice coefficient loss function—the ratio of the intersection of predicted and true labels over their average volume—was used to improve sensitivity to structure margins and normalize the weight of each classification region: left kidney, right kidney, and background. Each convolution layer utilizes filters with dimensions of 3 × 3 × 3 followed by batch normalization (20) and rectified linear unit activation layers (21). Following convolution at each resolution a 2 × 2 × 2 max pooling layer was used to downsample deeper network layers. After four convolution, normalization, activation, and max pooling stages, the network employs a similar process to upsample the native image resolution. The output of the activation layers prior to max pooling are concatenated with the output of the upsampled activation values of the same resolution using the U-Net methodology described by Ronneberger et al. (6). The overall network framework is given in Figure 1.

**Figure 2:**
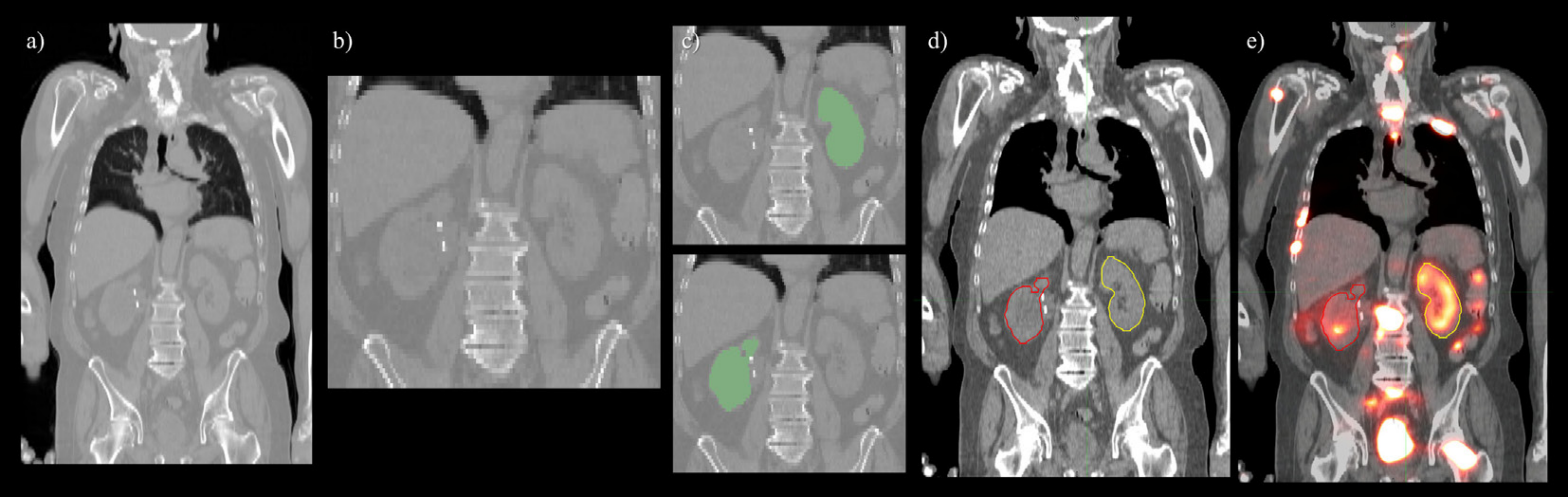
Pre- and post-processing workflow developed for automated renal dosimetry. Input image **(A)** is automatically cropped to a smaller search area based on alignment to a reference image volume. The cropped and downsampled image **(B)**, 128 × 128 × 64 voxels, is used as input to the convolutional neural networks segmentation model. Labeled left and right kidneys **(C)** are then upsampled, smoothed, and fused with the original, uncropped image **(D)**. Label map is exported in dicom-RT structure file, where voxel dose images may be analyzed on a hospital workstation **(E)**.

**Figure 3:**
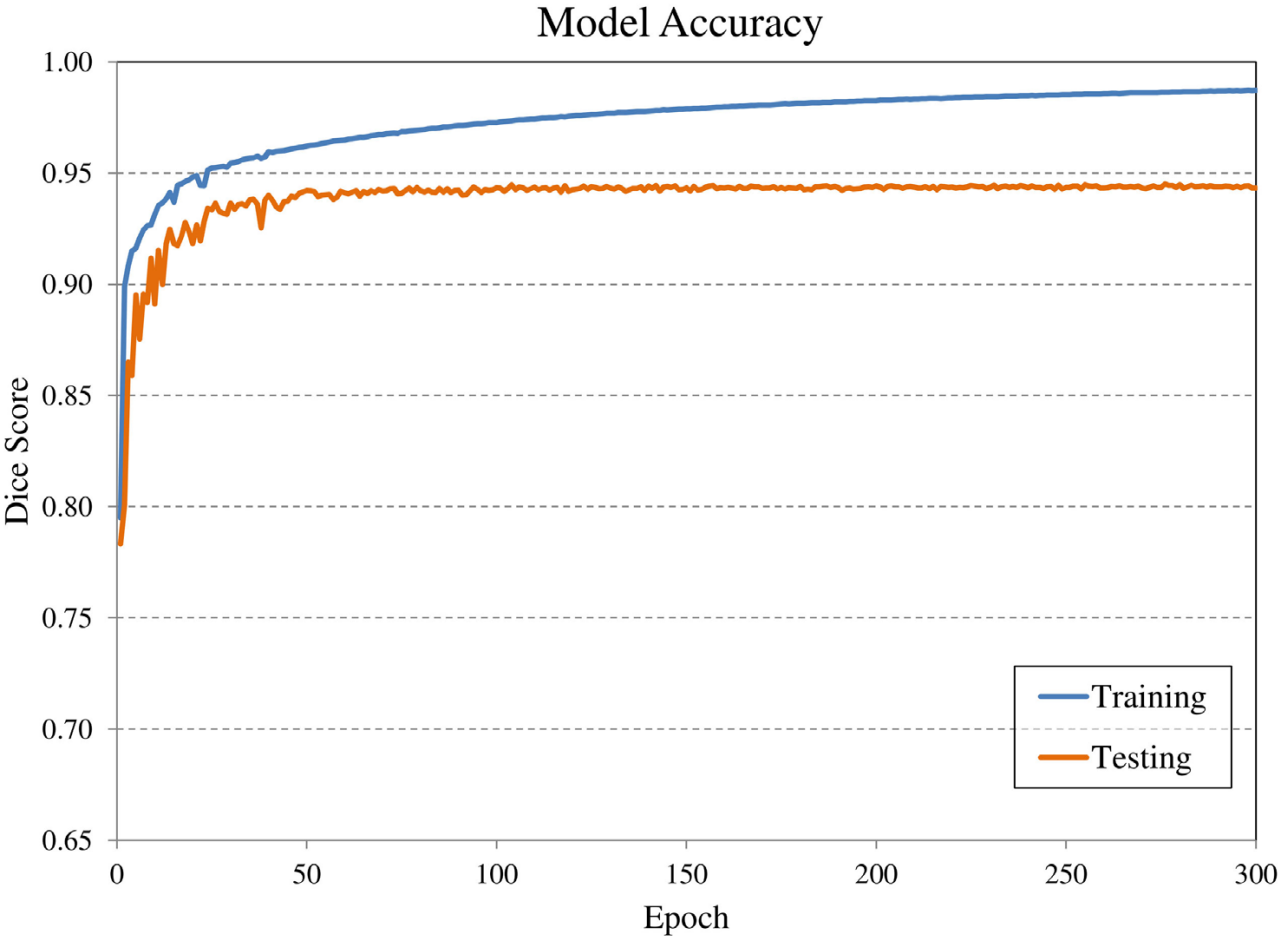
Evolution of model accuracy with over the 300 epochs for the training cohort (blue) and an augmented testing cohort (orange). Training required approximately 2.5 days for completion.

Convolutional neural network input volume is a matrix with dimensions 128 × 128 × 64 voxels. The workflow involved several pre-processing steps. First, bony anatomy was aligned with a reference patient by rigid registration (22). Images were cropped to a smaller search volume of 334 × mm x 334 × mm x 320 mm; a volume that could consistently capture the variation in kidney location between patients, while limiting the degree of downsampling required for input into the CNN algorithm. The native hybrid CT voxel resolution of 0.98 mm × 0.98 mm × 5.0 mm was subsequently resampled at 2.61 mm × 2.61 mm × 5.0 mm to achieve the required matrix dimensions. The complete workflow is illustrated in Figure 2. All training patients were pre-processed by the same methodology. Network training was allowed to run for 300 epochs using 640 teaching subjects. Another 72 augmented samples were used as a semi-independent scoring set to test training progress. Processing required 2.5 days on a cuda-enabled GPU (Nvidia GeForce GTX 1080 Ti) achieving dice accuracy of 0.98 with training data and 0.93 with a subset of augmented training patients as shown in Figure 3.

The software tool was integrated with the hospital PACS image database allowing selected CT studies to be transferred to a processing dicom node—a local computer—which returned the label map as a corresponding dicom-RT structure set. Structures could be viewed and modified on a standard imaging workstation and accessible hospital-wide. The process typically completed in less than 90 s; most of which was required for registration to the reference volume and post-processing to upsample the detected kidney labels at the original CT image resolution. CNNs contour detection required 10–15 s in most cases.

## Results

**Figure 4:**
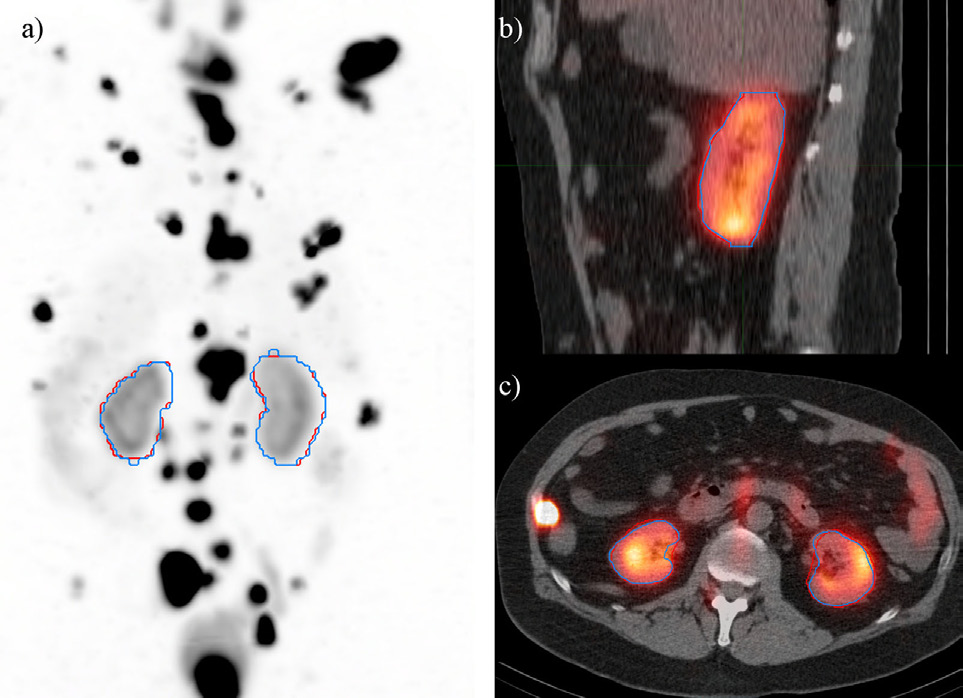
Typical case illustrating accuracy of the deep learning segmentation algorithm (red) in comparison to manual contours (blue). Contours are shown on **(A)** maximum intensity projection of voxel dose map, as well as **(B)** sagittal and **(C)** axial fused image sets.

**Figure 5:**
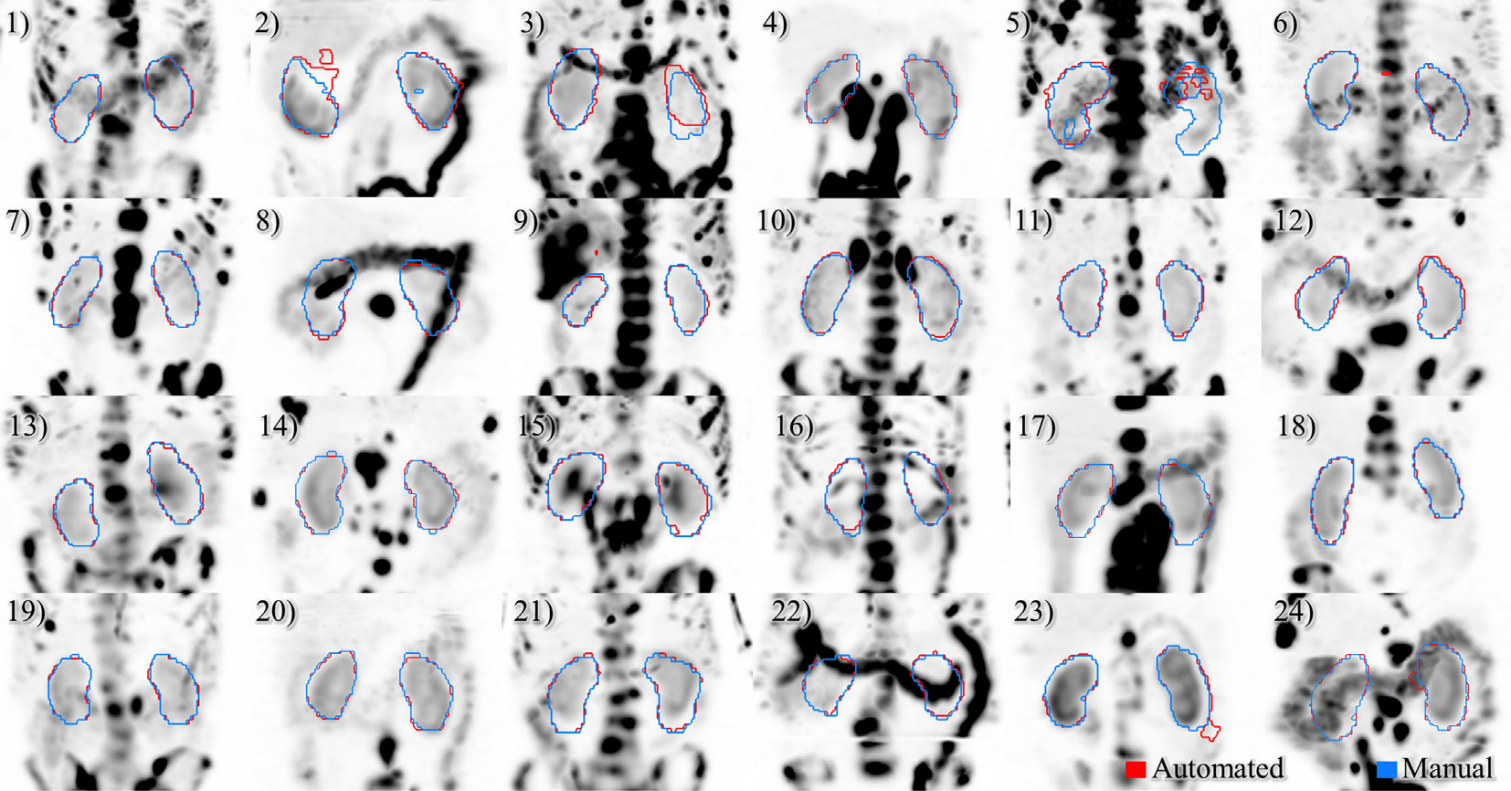
Results of automated (red) and manual (blue) segmentation overlaid with maximum intensity projections of voxel dose volumes. Patients 2 and 5 show some disagreement in cystic regions of kidney. In patients 6, 23, and 24 a small volume of bowel is captured by the automated algorithm.

A deep learning segmentation model was trained for detection and accurate delineation of kidneys on non-contrast, low-dose CT scans. A typical result overlaid on fused CT and voxel dose map is given in Figure 4. In more than 80% of cases, margins were in close visual agreement for both kidneys. Visual results of manual and automated contours overlaid with a coronal maximum intensity projection of the voxel dose map for each patient are shown in Figure 5. Even in poorly performing cases, some region of each kidney was detected with the developed registration and CNN method; a volume that was often representative of radionuclide uptake across the organ’s functional structure. When compared to manual segmentation as ground truth, automated contours achieved mean dice scores of 0.91 ± 0.05 and 0.86 ± 0.18 for right and left kidneys, respectively. The mean distance-to-agreement was estimated at 2.0 ± 1.0 and 4.0 ± 7.5 mm; a finer accuracy than the system resolution of typical SPECT imaging device.

Ignoring the one poorly performing left kidney with dice score of 0.11 and mean distance-to-agreement of 38.3 mm, left kidney accuracy is compared to the right side with a mean dice value of 0.89 ± 0.08 and MDA of 2.5 ± 1.7 mm. It should also be noted that the CNN-defined contours were consistently larger than those drawn manually by a factor of approximately 7%. This systematic effect likely attributed to the upsampling and smoothing of the predicted contours when returning to the native CT resolution and may be corrected by adjusting the prediction threshold to a value slightly above 0.5.

**Figure 6:**
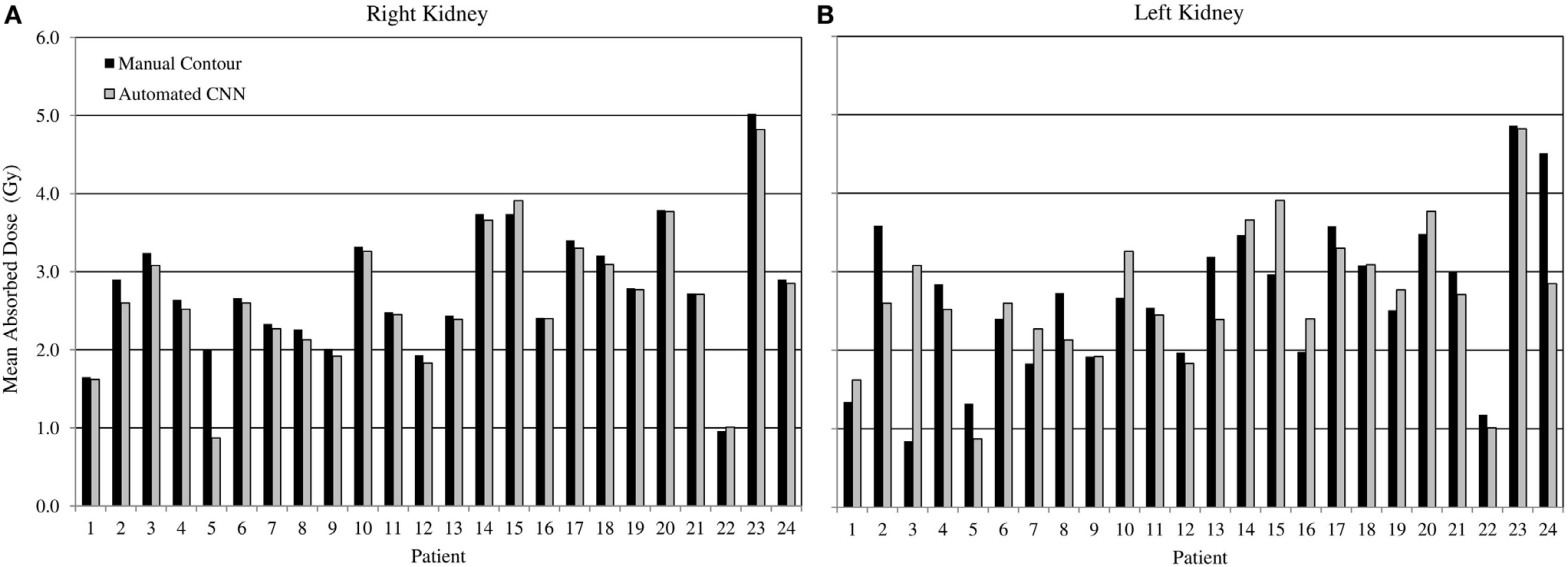
Estimated radiation absorbed dose applying either manual (black) or automated convolutional neural network segmentation (grey) to determine mean radiation absorbed to right and left kidneys from 3D voxel dose maps. Results for right **(A)** and left **(B)** kidneys are presented independently.

Comparing radiation dose estimates from automated and manually drawn contours, there is no apparent bias using either technique (Figure 6). Across the cohort there was an average difference in dose estimate of 3.0% in the right kidney and −3.6% in the left. SD of the error was ±4.5 and ±5.7%, respectively. If omitting the results for patients with cystic kidneys which would be reviewed and corrected in a clinical workflow—patients #2, 3, and 5 in Figure 5—the discrepancy in dose estimates between manual and automated methods is less than 2% for both kidneys. Based on *t*-test of null hypothesis, no difference between dose estimates between groups was detected (*p* = 0.03 and *p* = 0.01, right and left). Results of contour accuracy and renal radiation dose for each patient are reported in Table S1 in Supplementary Material.

**Figure 7:**
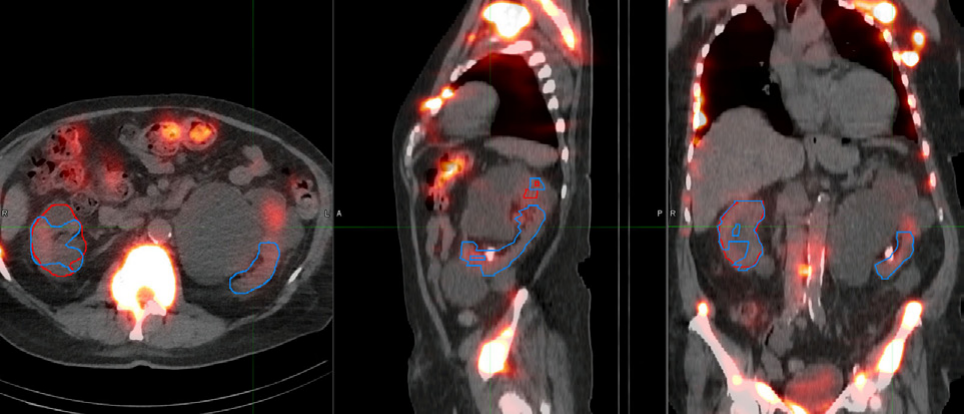
Most challenging case encountered in testing the renal convolutional neural networks. Due to multiple large cysts in originating within the central renal structure, the segmentation tool detected only 20 cc of the manually contoured 167 cc left kidney volume (dice = 0.11).

Three of the patients in the ^177^Lu-PSMA therapy cohort displayed highly cystic kidneys; to a degree that was not observed in the training patients (Figure 7). In these cases, the mean dice score was dramatically lower at 0.66. No systematic increase or decrease in estimated dose was shown (−2.70%) indicating that often the CNN-contoured region was representative of the mean uptake in the manually delineated kidney. In another three patients, a small, detached section (<10 cc) of bowel was included one of the contours. In none of the cases did error manifest in an appreciable effect on estimated renal dose. If frequently noted, small non-contiguous labels could be detected and removed as a post-processing step. Only one patient with structurally normal kidneys showed poor performance with the segmentation algorithm omitting approximately one-third of the left kidney volume (dice = 0.67); an error which coincided with a region of CT streak artifact.

## Discussion

The advent or rapid, accurate tissue contouring through deep learning segmentation demonstrates the potential for quantitative diagnosis in molecular imaging. In this study, the results of a CNN trained to detect kidneys on CT images have been used to assess regional radiation exposure in unsealed source therapy. In principle, contouring of tumors and at-risk tissues is the last remaining step in nuclear medicine dosimetry that required manual oversight. We have combined automated kidney segmentation with a previous work that computed voxel dose maps from serial post-treatment SPECT images to demonstrate the feasibility of a fully automated system. The time required to process a case with manual methods may require several hours and may be subjected to systematic variability due to the method of curve fitting and drawn contour margins.

While the automated system performs well in most cases—achieving dice scores which are comparable to inter-observer variability between manual scores in CT (23)—it is advisable to review all contours before being relied upon for quantitative assessment. In this instance, the training cohort was not necessarily representative of the patients used for testing. Those used to train the model were generally younger, from both genders, and did not include cases with cystic kidneys which were observed in 3 of the 24 testing cases. In the preparation of this framework, considerable improvement was noted over multiple iterations of the renal CNN as challenging cases were flagged, manually contoured, and incorporated into subsequent training files. It is worth noting that the addition of these irregular patients did not hinder the accuracy of the CNN when detecting otherwise normal anatomy. From the experience in developing this tool, the authors speculate that features which would accommodate detection of functional regions in polycystic kidneys would develop as the model which was retrained with additional poorly performing cases.

Previous methods such as the one described by Hasegawa do appear sound for segmentation of two-dimensional images (24). The majority of recent publications involving semantic segmentation employ variations on the U-Net structure described by Ronneberger et al. (6). These have been adapted to 3D image volumes and have proven sufficiently accurate to avoid the need for shape-based post-processing. The depth of these networks may be considered overkill when comparing the complexity of the segmentation task relative to the number of parameters that define the model weights. However, the computational requirements to train and apply such a model are feasible on a standard PC and they (or slight variations) have been shown to be extremely adaptable to a multitude of image segmentation tasks (13, 14, 25, 26). In the present work, we have, therefore, chosen to adopt the CNN approach given that a more complex algorithm may also prove more adaptable with issues concerning some of the more complex structural abnormalities, such as renal cysts.

By employing a dice score loss function based on the accuracy of trained kidney margins rather than the total number of correctly categorized voxels, a dramatic improvement in the detection of kidney margins was observed. In the former version, as employed by Pazhitnykh et al. to contour lungs (18), the model was heavily weighted to correctly designate background (non-label) voxels which typically comprised more than 90% of the search volume. In this initial iteration, the CNN could be trained to routinely identify some or the majority of kidney tissue, but was not sensitive to small boundary errors because these only manifest in subtle changes to the overall accuracy calculation. The combination of dice score and training data augmentation greatly improved the algorithm utility; correctly identifying organ margins in approximately 80% of cases. The model reported in this work was further improved by the addition of challenging cases that were flagged as poorly delineated by the existing CNN. This method could be applied to other challenging soft tissue regions and hope to implement a more comprehensive set of organs in future nuclear medicine dosimetry tools. For smaller organs or tumors, it may be advisable to utilize a tighter search volume or sliding window technique to perform classification at or near the native CT image resolution. There is also the potential to feed the fused SPECT/CT or PET/CT dataset into the CNN, capitalizing on complimentary features in both image domains to improve specificity.

## Conclusion

Medical image segmentation by CNNs shows merit in the analysis of post-treatment scans in order to practically estimate radiation dose from unsealed source therapies. Deep learning methods have been applied to consistently detect right and left kidneys with no significant difference between radiation dose determined from CNN contours compared with manual methods. The tool has been combined with a previously developed voxel dose processing technique demonstrating the potential for fully automated radiation dose estimation for nuclear medicine therapies in the near future.

## Availability of Data and Materials

The patient datasets used in this study are not available to the public. The neural network model as well as pre- and post-processing computer software may be distributed on request to the corresponding author.

## Ethics Statement

^177^Lu-PMSA-617 trial was approved by the institutional ethics board and registered with the Australian New Zealand Clinical Trials Registry (ANZCTR12615000912583). The study protocol was conducted in accordance with the Declaration of Helsinki and Good Clinical Practice and all patients gave written informed consent prior to entry on the study.

## Author Contributions

PJ developed the image processing techniques described in this research article. NH assisted with the similarity analysis of contour shape and radiation absorbed dose comparison. ND provided assistance with development of neural network software in Python. TK helped with study design and guided practical implementation as a hospital tool. MH was clinical lead on 177Lu-PSMA therapy trial and with the assistance of RH provided access to validation images used in this study. All authors contributed to the review and authorization of the paper.

## Conflict of Interest Statement

The authors declare that the research was conducted in the absence of any commercial or financial relationships that could be construed as a potential conflict of interest.
