# Supplementary material for: Deep Learning Renal Segmentation for Fully Automated Radiation Dose Estimation in Unsealed Source Therapy
Source: Front Oncol. 2018 Jun 14;8:215. doi: 10.3389/fonc.2018.00215 (PMC6010550; doi:10.3389/fonc.2018.00215)
Supplement: Supplementary file 1 [file Data_Sheet_1.docx]

**Appendix:**

Image Augmentation:

Variable augmentations were applied by applying random image rotations, translations, Gaussian smoothing, change in global Housfield value, added noise, and edge enhancement. Each parameter could be programmatically adjusted through python script across a maximum range of values that would permit realistic variations in image appearance. Image rotation was applied in the axial plane with a maximum degree value of ±7.5°. A random of translation up to 5 voxels was applied along each axis. Three-dimensional Gaussian smoothing was applied with a maximum sigma value of 0.5. Global Housfield value shift was designated up to ±7.5 HU. Noise was added with a value of ±15 HU. Lastly a random degree of edge enhancement was applied by subtracting the original image from a Gaussian blurred image (sigma range 0.35 to 0.48) with an opacity value, *α*, ranging from 0.18 to 0.42. The results of the random augmentations applied to a single image series are illustrated below.


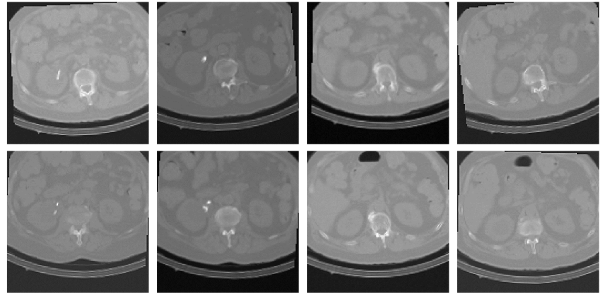


Figure 8. Illustration of training image volume following augmentation by random rotation, translation, added noise, smoothing, and edge enhancement. Augmentation was used to increase the quantity of training data by a factor of eight.

|  | Right Kidney | |  |  |  |  | Left Kidney | |  |  |  |  |
| --- | --- | --- | --- | --- | --- | --- | --- | --- | --- | --- | --- | --- |
| Patient # | Dice Coefficient | MDA (mm) | Volume (cc, manual) | Volume (cc, CNN) | Dose (Gy, manual) | Dose (Gy, CNN) | Dice Coefficient | MDA (mm) | Volume (cc, manual) | Volume (cc, CNN) | Dose (Gy, manual) | Dose (Gy, CNN) |
| 1 | 0.930 | 1.44 | 151.2 | 152.7 | 1.65 | 1.62 | 0.914 | 1.90 | 177.6 | 202.9 | 1.34 | 1.29 |
| 2 | 0.838 | 4.64 | 227.0 | 269.9 | 2.90 | 2.60 | 0.794 | 4.48 | 231.9 | 345.1 | 3.59 | 3.03 |
| 3 | 0.879 | 2.80 | 212.8 | 257.8 | 3.24 | 3.08 | 0.627 | 7.70 | 111.0 | 143.7 | 0.84 | 0.76 |
| 4 | 0.906 | 2.00 | 142.6 | 147.0 | 2.64 | 2.52 | 0.894 | 2.44 | 167.1 | 195.8 | 2.84 | 2.69 |
| 5 | 0.713 | 4.95 | 190.9 | 286.5 | 2.00 | 0.87 | 0.108 | 38.32 | 163.5 | 19.9 | 1.32 | 1.02 |
| 6 | 0.921 | 2.22 | 184.8 | 206.5 | 2.66 | 2.60 | 0.943 | 1.28 | 185.3 | 192.7 | 2.40 | 2.39 |
| 7 | 0.920 | 1.59 | 142.4 | 156.0 | 2.33 | 2.27 | 0.911 | 1.86 | 164.3 | 187.3 | 1.83 | 1.73 |
| 8 | 0.905 | 2.13 | 201.5 | 234.8 | 2.26 | 2.13 | 0.916 | 2.02 | 204.0 | 227.5 | 2.73 | 2.67 |
| 9 | 0.868 | 2.29 | 75.3 | 87.3 | 2.01 | 1.92 | 0.874 | 2.33 | 105.2 | 127.7 | 1.92 | 1.79 |
| 10 | 0.934 | 1.32 | 176.9 | 187.4 | 3.32 | 3.26 | 0.903 | 1.79 | 182.7 | 197.4 | 2.67 | 2.71 |
| 11 | 0.945 | 1.21 | 186.6 | 193.8 | 2.48 | 2.45 | 0.933 | 1.47 | 183.1 | 198.0 | 2.54 | 2.46 |
| 12 | 0.903 | 2.30 | 191.6 | 221.9 | 1.93 | 1.83 | 0.896 | 2.37 | 209.0 | 241.0 | 1.97 | 1.92 |
| 13 | 0.931 | 1.48 | 156.0 | 168.6 | 2.44 | 2.39 | 0.918 | 1.88 | 211.9 | 233.6 | 3.19 | 3.26 |
| 14 | 0.934 | 1.41 | 159.9 | 173.6 | 3.74 | 3.66 | 0.935 | 1.44 | 153.5 | 166.5 | 3.47 | 3.41 |
| 15 | 0.922 | 2.06 | 259.7 | 249.3 | 3.74 | 3.91 | 0.671 | 5.64 | 277.3 | 153.7 | 2.97 | 3.11 |
| 16 | 0.916 | 1.55 | 121.3 | 128.2 | 2.41 | 2.40 | 0.932 | 1.30 | 125.2 | 133.9 | 1.98 | 1.94 |
| 17 | 0.944 | 1.21 | 152.3 | 160.5 | 3.40 | 3.30 | 0.955 | 1.02 | 175.5 | 180.0 | 3.58 | 3.54 |
| 18 | 0.933 | 1.34 | 177.9 | 193.9 | 3.21 | 3.09 | 0.932 | 1.47 | 173.2 | 186.2 | 3.08 | 3.02 |
| 19 | 0.938 | 1.28 | 150.3 | 151.7 | 2.79 | 2.77 | 0.936 | 1.49 | 185.3 | 196.9 | 2.51 | 2.47 |
| 20 | 0.919 | 1.66 | 134.9 | 145.0 | 3.79 | 3.77 | 0.907 | 1.99 | 162.6 | 178.9 | 3.48 | 3.43 |
| 21 | 0.943 | 1.29 | 190.8 | 200.0 | 2.72 | 2.71 | 0.934 | 1.53 | 214.7 | 223.1 | 3.01 | 3.03 |
| 22 | 0.917 | 1.76 | 162.2 | 150.8 | 0.96 | 1.01 | 0.912 | 1.87 | 142.3 | 158.2 | 1.18 | 1.14 |
| 23 | 0.924 | 1.85 | 207.9 | 233.1 | 5.02 | 4.82 | 0.935 | 3.11 | 228.9 | 252.6 | 4.86 | 4.63 |
| 24 | 0.942 | 1.55 | 311.3 | 330.8 | 2.90 | 2.85 | 0.927 | 5.55 | 266.1 | 285.0 | 4.51 | 4.47 |
| Mean | 0.909 | 1.97 | 177.8 | 195.3 | 2.77 | 2.66 | 0.859 | 4.01 | 183.4 | 192.8 | 2.66 | 2.58 |
| Standard Deviation | 0.049 | 0.96 | 47.3 | 56.6 | 0.84 | 0.90 | 0.179 | 7.49 | 42.7 | 60.9 | 1.01 | 1.00 |

Table 1. Results of contour overlap and radiation absorbed dose for each ^177^Lu-PSMA patient analysed in this study.
